# Thermal and Rheological Characterization of Recycled PET/Virgin HDPE Blend Compatibilized with PE-g-MA and an Epoxy Chain Extender

**DOI:** 10.3390/polym14061144

**Published:** 2022-03-12

**Authors:** Raquel M. Santos, Anna R. M. Costa, Yêda M. B. Almeida, Laura H. Carvalho, João M. P. Q. Delgado, Elisiane S. Lima, Hortência L. F. Magalhães, Ricardo S. Gomez, Boniek E. Leite, Fagno D. Rolim, Maria J. Figueiredo, Antonio G. B. Lima

**Affiliations:** 1 Postgraduate in Process Engineering, Federal University of Campina Grande, Campina Grande 58429-900, Brazil; raquelmarques_santos@outlook.com (R.M.S.); boniek3@gmail.com (B.E.L.); 2 Department of Chemical Engineering, Federal University of Pernambuco, Recife 50740-520, Brazil; raffaela_matos@yahoo.com.br (A.R.M.C.); yeda.oliveira@ufpe.br (Y.M.B.A.); 3 Department of Materials Engineering, Federal University of Campina Grande, Campina Grande 58429-900, Brazil; heckerdecarvalho@yahoo.com.br; 4 CONSTRUCT-LFC, Department of Civil Engineering, Faculty of Engineering, University of Porto, 4200-465 Porto, Portugal; 5 Department of Mechanical Engineering, Federal University of Campina Grande, Campina Grande 58429-900, Brazil; limaelisianelima@hotmail.com (E.S.L.); ricardosoaresgomez@gmail.com (R.S.G.); antonio.gilson@ufcg.edu.br (A.G.B.L.); 6 Department of Chemical Engineering, Federal University of Campina Grande, Campina Grande 58429-900, Brazil; hortencia.luma@gmail.com; 7 Teacher Training Center, Federal University of Campina Grande, Cajazeiras 58900-000, Brazil; dallino@hotmail.com; 8 Department of Agro-Industrial Management and Technology, Federal University of Paraíba, Bananeiras 58220-000, Brazil; mariaufp@gmail.com

**Keywords:** PET, HDPE, PE-g-MA, joncryl, torque rheometry, thermal properties

## Abstract

In this work, recycled poly(ethylene terephthalate) (PETR) was blended with virgin high-density polyethylene (HDPE) in an internal mixer in an attempt to obtain a material with improved properties. A compatibilizer (PE-g-MA) and a chain extender (Joncryl) were added to the PETR/HDPE blend and the rheological and thermal properties of the modified and unmodified blends as well as those of virgin PET with virgin HDPE (PETV/HDPE). All the blends were characterized by torque rheometry, differential scanning calorimetry (DSC) and thermogravimetric analysis (TGA). The data obtained indicate that the incorporation of either the chain extender or the compatibilizer agent led to increases in torque (and hence in viscosity) of the blend compared to that of the neat polymers. The joint incorporation of the chain extender and compatibilizer further increased the viscosity of the systems. Their effect on the crystallinity parameters of HDPE was minimal, but they reduced the crystallinity and crystallization temperature of virgin and recycled PET in the blends. The thermal stability of the PETR/HDPE blend was similar to that of the PETV/HDPE blend, and it was not affected by the incorporation of the chain extender and/or compatibilizer.

## 1. Introduction

Poly(ethylene terephthalate) (PET), the most common thermoplastic polymer resin of the polyester family, has become one of the main contributors to post-consumer plastic waste. Its properties and low cost are responsible for its high production, which in turn has led to serious environmental problems as most of the products manufactured with this resin are fast disposal products which growingly accumulate in landfills [1].

Many efforts are directed towards improving methods for recycling and reusing plastic components from industrial and municipal waste. However, the costs for processing recycled polymers are often not competitive with those of virgin products. Blending post-consumer polymers under suitable conditions can provide an alternative route to the commercialization of recycled materials with a satisfactory cost/performance ratio and application potentials in packaging and the household and engineering sectors [2].

PET and polyolefins (PO) such as high-density polyethylene (HDPE), low density polyethylene (LDPE) and polypropylene (PP) are the most widely used thermoplastics in packaging applications (bottles, containers, films, etc.) which have a low shelf life [3]. HDPE has high impact strength, while PET has excellent heat resistance, hardness and chemical stability. It is believed that PET/HDPE blends can be particularly advantageous, as new materials with promising property combinations can be obtained at relatively low costs. In addition, these blends can be manufactured with recycled materials, adding value to the waste.

However, the production of artifacts from PET/HDPE blends is a challenging task, as they are immiscible and the properties of their non-compatibilized blends are unsatisfactory [4]. In addition, the presence of impurities such as adhesives, pigments, metals, as well as different types of incompatible polymer components, affect the properties of recycled plastic materials. In general, the result is poor dispersion of the components and low interfacial adhesion, which negatively affect the physical–mechanical properties of these materials [5].

Several studies on the compatibilization of PET and PO blends have been published [6,7,8,9,10]. The most commonly used reactive agents or functional groups for the compatibilization of PET with PO are acrylic acid, maleic anhydride and epoxy groups, which react with the carboxyl or hydroxyl end groups of PET [4,5,11,12]. Uehara et al. [13] studied PET/HDPE blends from recycled leftover multilayer packaging films compatibilized with maleic anhydride (PE-g-MA) and glycidyl methacrylate (PE-GMA) and concluded that PE-g-MA proved to be the best additive for the compatibilization of that blend. Pracella et al. [14] published a review on the properties of PET/PO blends and showed that compatibilization with various functionalized polyolefins allowed for greater phase dispersion, reduced interfacial tension and improved interfacial adhesion. Taghavi et al. [15] stated that maleic anhydride grafted polyethylene (PE-g-MA) provides better bonding between recycled poly(ethylene terephthalate) and HDPE compared to maleic anhydride grafted ethylene/butylene–styrene copolymer (SEBS-g-MA). Chen [16] reported that LDPE/PP/PET blend compatibilized with maleic anhydride grafted polyolefins showed improved thermal stability, increased elongation at break and decreased tensile strength.

Harth et al. [17] compared Joncryl ADR 4368 and pyromellitic dianhydride (PMDA) to improve the melt properties, in order to allow film blowing. The study showed that both modifiers are able to increase the melt strength at low concentrations of 0.1 up to 0.4 wt. % but that Joncryl is more efficient in terms of the generation of long-chain branched molecules. Wu et al. [18] found five chain extenders with different numbers of epoxy groups per molecule that were used to mix with discarded PET fibers and improve their viscosity and quality loss in the recycling process. All PET samples modified by Joncryl ADR-4468 with about 9–15 epoxy groups have been proven to have higher viscosities and better thermal properties, exhibiting a long-chain branched structure and better crystallization. Nofar et al. [19] also examined the use of Joncryl in blends of PET/PBT that are miscible in their amorphous phases, while the miscibility of their crystalline phase varies depending on the cooling rate. It was verified that Joncryl mainly increased the melt viscosity of PET and its blend with PBT and, at the low contents, increased the PET’s crystallization rate. The strain-at-break values of the blends were also increased with the use of only 0.2 wt. % Joncryl.

The molar mass of PET decreases during melt processing due to high temperatures and hydrolytic degradation, an effect enhanced during processing of recycled PET due to the presence of contaminants that act as catalysts for hydrolytic degradation [20]. Therefore, to minimize this problem and to compensate for thermal, oxidative and/or hydrolytic degradation during processing, chain extenders are added to PET in order to recover or to increase its molar mass during processing and to act as a reactive compatibilizer for polymer blends [19,20,21,22,23,24,25,26]. Duarte et al. [26] studied chain extension during processing and reprocessing of virgin and recycled PET and showed that levels of 0.5 and 1.0% of the multifunctional epoxy oligomer (Joncryl ADR 4368) were sufficient to compensate for sample degradation. Nofar et al. [19] reported that the addition of the commercial chain extender additive (Joncryl ADR 4468) in poly(butylene) terephthalate/recycled poly(ethylene terephthalate) blends increased melt viscosity. However, additive contents above 0.8% delayed recycled poly(ethylene terephthalate) crystallization, while 0.4% content had an opposite effect, acting as a nucleating agent, increasing the crystallization rate of recycled poly(ethylene terephthalate).

Considering the above, the need for improvements in recycling processes and their importance to the environment, it becomes necessary to study and develop new methods of processing these wastes and to enable new applications. So far, there are reports on the compatibilization of PET/HDPE blends with chain extenders based on epoxy compounds (Joncryl) and their joint action with PE-g-Ma. It is important to investigate this system for the possible reuse of these polymers.

Therefore, the objective of this work is to evaluate the effect of a chain extender (Joncryl) and its combination with a compatibilizer (PE-g-MA), on the characteristics of PET/HDPE blends obtained from post-consumer and virgin PET (PETR and PETV) and virgin HDPE, with respect to their rheological and thermal properties.

## 2. Methodology

### 2.1. Materials

The high-density polyethylene (HDPE) JV060U, with 0.957 g/cm^3^ and MFI of 7.0 g/10 min, was supplied by Braskem (Maceió, Brazil) [27]. DSC data revealed that it melts between 105 and 140 °C, with a peak temperature at 134 °C [28].

The virgin poly(ethylene terephthalate) (PETV), brand name Cleartuf Turbo, was supplied by M&G Polyester (São Paulo, Brazil). According to the manufacturer, this material has an intrinsic viscosity of 0.8 dL/g, density of 1.39 g/cm^3^ and melting point of 246 °C [29]. The post-consumer recycled PET (PETR) used in this study came from colorless soft drink containers (bottles) collected in the state of Paraíba and was supplied as flakes by the company DEPET/PB (Campina Grande, Brazil).

Maleic anhydride grafted polyethylene (PE-g-MA) trade name Polybond 3009 with 1% maleic anhydride, 0.95 g/cm^3^ density, melt flow index of 5.0 g/10 min and melting temperature of 127 °C [30] was supplied by Chemtura (Rio Claro, Brazil) and used as a compatibilizer for PETV/PEAD and PETR/PEAD blends. The amount of PET-g-MA employed in each blend was 10% *w*/*w* as suggested in the literature [28].

The multifunctional epoxy additive Polyad PR 002 (a blend of Joncryl 4368 and Joncryl 4370), supplied by BASF (São Paulo, Brazil), was used as a chain extender for PET and, in this paper, will be called “Joncryl”. With a weight average molar mass of 6800 g/mol, this additive contains 4 to 10 units of epoxy groups per molecule [31,32]. The amount of chain extender used in the blends was 1% *w*/*w*, an amount sufficient to promote molar mass recovery [20].

### 2.2. Experimental Procedures

#### 2.2.1. Blend Processing

PETV and PETR were oven dried before processing at 130 °C for 6 h. HDPE, compatibilizer and chain extender were used as received. All samples (neat polymers and their blends with and without the additives) were processed in Thermo Scientific Haake Rheomix 3000 (Thermo Fisher Scientific, São Paulo, Brazil) internal mixer fitted with high-intensity roller-type rotors operating at 60 rpm (N) for 15 min (t_p_) and 265 °C wall temperature (T_0_).

Pure polymers and the neat blends were processed and the average values of three processing runs are presented here.

Virgin and recycled PET containing 1% *w*/*w* Joncryl were coded PETVJ and PETRJ, respectively. Joncryl was not added to neat HDPE as it is a polyester-specific additive, which should be inert with polyolefins.

Pristine, additivated, compatibilized as well as additivated and compatibilized blends of PET (virgin or recycled)/HDPE with 1:3; 1:1 and 3:1 mass ratios were manufactured. Additivated compositions had 1 phr Joncryl; compatibilized compositions had 10 phr PE-g-MA and additivated and compatibilized compositions had 1 phr Joncryl and 10 phr PE-g-MA.

Pristine blends containing virgin or recycled PET were coded BLV x/y and BLR x/y, respectively. Compatibilized blends with virgin and recycled PET were coded BLVM x/y or BLRM x/y. Compatibilized and additivated blends were coded BLVMJ x/y or BLRMJ x/y, respectively. In all cases, x and y refer to the mass ratio of PET (PETV or PETR) and HDPE in the blend.

The systems investigated are listed in Table 1.

#### 2.2.2. Rheological Characterization

Analysis of temperature T(t) and torque Z(t) as functions of processing time at the last stages of melt processing allows us to estimate the rheological characteristics (viscosity dependence on temperature and shear rate) of the processed material and to evaluate incipient degradation rate during processing [33,34,35,36,37].

During the last stage of processing of polymeric systems (melt), torque *Z* is proportional to the melt viscosity that, in turn, depends exponentially on the temperature of the molten polymer inside the laboratory mixer, as:

(1) $$\eta =kexp\left\{-\beta \left(T-T^{*}\right)\right\}\;$$

where *T* is the melt temperature, $T^{*}$ is an arbitrary reference temperature and *β* is the viscosity temperature coefficient [35]. Therefore:

(2) $$Z=kexp\left\{-\beta \left(T-T^{*}\right)\right\}\;$$

or

(3) $$lnZ=lnk-\beta \left(T-T^{*}\right)\;$$

During processing of molten polymers in the internal mixer, there is predominantly shear flow. The deformation rate can be associated with shear rate, which depends on rotor speed (N) and the geometry of the equipment. For a fluid whose rheological characteristics can be represented by the power law, torque is given by:

(4) $$Z=k_{1}N^{n}exp\left\{-\beta \left(T-T^{*}\right)\right\},$$

where k is a constant for tests performed in the same apparatus and processing conditions, i.e., the same mixer/rotors combination, fill factor and rotor speed; N is the rotation speed, and n is the pseudoplasticity index.

The temperature effect can be eliminated by setting the adjusted torque $Z^{*}$ to the reference temperature $T^{*}$ as follows:

(5) $$Z^{*}=Zexp\left\{+\beta \left(T-T^{*}\right)\right\}.$$

For the adjusted torque, Equation (4) takes the form:

(6) $$Z^{*}=k_{1}N^{n},$$

or

(7) $$lnZ^{*}=lnk_{1}+nlnN.$$

By using Equations (2) and (6), it is possible to evaluate the rheological parameters *β* and *n*, respectively, from temperature and torque values at the end of processing. This is accomplished from two series of experiments:

(a) Tests conducted at constant rotor rotation speed (N) and different processing chamber wall temperatures (*T*), which allow the determination of *β* by linear regression of $ln\bar{Z}$ versus $\bar{T}-T^{*}$, where $\bar{Z}$ and $\bar{T}$ are average torque and temperature values and $T^{*}$ an arbitrary reference temperature, and
(b) Tests conducted at constant processing chamber wall temperature (T) and different rotor rotation speeds (N), which allow calculation of the parameter n by linear regression of $ln\bar{Z}$ versus lnN.

Three tests were performed at different processing chamber wall temperatures (265, 280 and 290 °C) to obtain the temperature coefficient of viscosity *β* of the PETV/PEAD blend (1:1). Three other tests were performed at different rotor speeds (30, 60 and 120 rpm), keeping the chamber wall at a temperature of 265 °C, to obtain the pseudoplasticity index n for the same samples.

Degradation and recovery during processing.

Polymers may either thermally degrade or crosslink during processing, which changes their viscosity, and hence torque, during processing. Melt temperature also varies with composition and processing conditions and since torque and viscosity are strongly temperature dependent, in order to compare results, it is necessary to eliminate the effect of temperature on torque, so that only the effects of molar mass variation over time are observed. In order to do this, torque data is adjusted to a common reference temperature $T^{*}$ using the temperature coefficient (*β*) previously calculated. The adjusted torque $Z^{*}$ is then obtained as

(8) $$Z^{*}=Zexp\left\{+\beta \left(T-T*\right)\right\}.$$

The expressions above assume stable polymeric resins, that is, that do not degrade and whose molar masses do not change during processing. However, most polymers gradually degrade during processing at moderately high temperatures, resulting in a drop in their average molar mass. It is demonstrated that, for a fluid whose rheological characteristics can be represented by the power law, the “constant” *k* of Equation (2) is a function of the weight average molar mass Mw [38], and then:

(9) $$k\approx k{}^{\prime }M_{w}^{2.5+n}.$$

Taking into account the definition of adjusted torque, Equation (9), we obtain:

(10) $$Z^{*}\approx k{}^{\prime }M_{w}^{2.5+n}.$$

Therefore, the relative rate of change of the adjusted torque at the final stage of processing will be given by:

(11) $$R_{Z}=\frac{1}{\bar{Z^{*}}}\frac{dZ^{*}}{dt{}^{\prime }}$$

which is a measure of the rate of degradation ($R_{Z}$ < 1) or chain length increase ($R_{Z}$ > 1).

The value of $R_{Z}\;$× 100 represents the percentage of torque variation per unit of time at the final stage of processing under a given set of experimental conditions (T, rpm, rotor geometry and chamber temperature).

$R_{Z}$ was determined for different samples using the *β* value estimated for the PET/HDPE blend (50/50) and the adjusted torque $Z^{*}$(t) estimated in a small time interval at the final stage of processing. The mean value of $Z^{*}$ was determined and then d$Z^{*}$/dt was estimated by linear regression of $Z^{*}$ versus time.

If the pseudoplasticity index n is known, the rate of change of the weight average molar mass can be estimated as follows:

(12) $$R_{M}=\frac{1}{\bar{M_{w}}}\frac{dM_{w}}{dt}=\frac{1}{2.5+n}R_{Z}.$$

#### 2.2.3. Differential Scanning Calorimetry

Differential scanning calorimetry (DSC) analyses were performed on a Mettler Toledo DSC-1 instrument (Barueri, Brazil) using a standard aluminum crucible with a volume of 40 μL. The tests were carried out under nitrogen flow rate of 50 mL/min and 5 to 7 mg samples. PET and PET/HDPE blend samples were investigated using a three-stage thermal program: heating from 20 to 280 °C, cooling to 20 °C and reheating to 280 °C. The HDPE samples were analyzed using a similar thermal program but with the maximum temperature set at 200 °C. The heating/cooling ratio for all samples was 10 °C/min.

The latent heat of crystallization/melting per unit mass of the crystallizable polymer was determined as:

(13) $$\Delta H=\frac{E_{0}}{w_{P}m_{S}{}^{\prime }}$$

where $m_{S}$ is the sample mass and $w_{P}$ is the mass fraction of the polymer under analysis. The change in crystallinity during the event is given by:

(14) $$\Delta X=\frac{\Delta H}{\Delta H_{m}^{0}{}^{\prime }}$$

where $\Delta H_{m}^{0}$ is the latent heat of melting of the 100% crystalline polymer. $\Delta H_{m}^{0}$ values of 293 J/g and 145 J/g for HDPE and PET, respectively, are provided in the literature [36,37]. In this work, it was assumed that both the HDPE and the compatiblizer (PE-g-MA) crystallize and melt at the same temperature, and that their limit latent heats are equal; therefore, in the compatibilized blends, the weight fraction $w_{P}$ in Equation (13) was considered to be the sum of the fractions of HDPE and PE-g-MA.

#### 2.2.4. Thermogravimetry

Thermogravimetric analyses (TGA) were performed on a Shimadzu DTG-60H instrument, using alumina crucible under nitrogen atmosphere, with sample mass between 10 and 12 mg. Samples were heated at a rate ϕ = 10 °C/min from 30 °C to 700 °C under inert atmosphere (100 mL/min nitrogen gas). Samples of all PET/HDPE blends and the components HDPE, PETV and PETR, as well as the compatibilizer (PE-g-MA) and the additive (Joncryl) were analyzed.

## 3. Results and Discussion

### 3.1. Rheological Characterization

**Figure 1:**
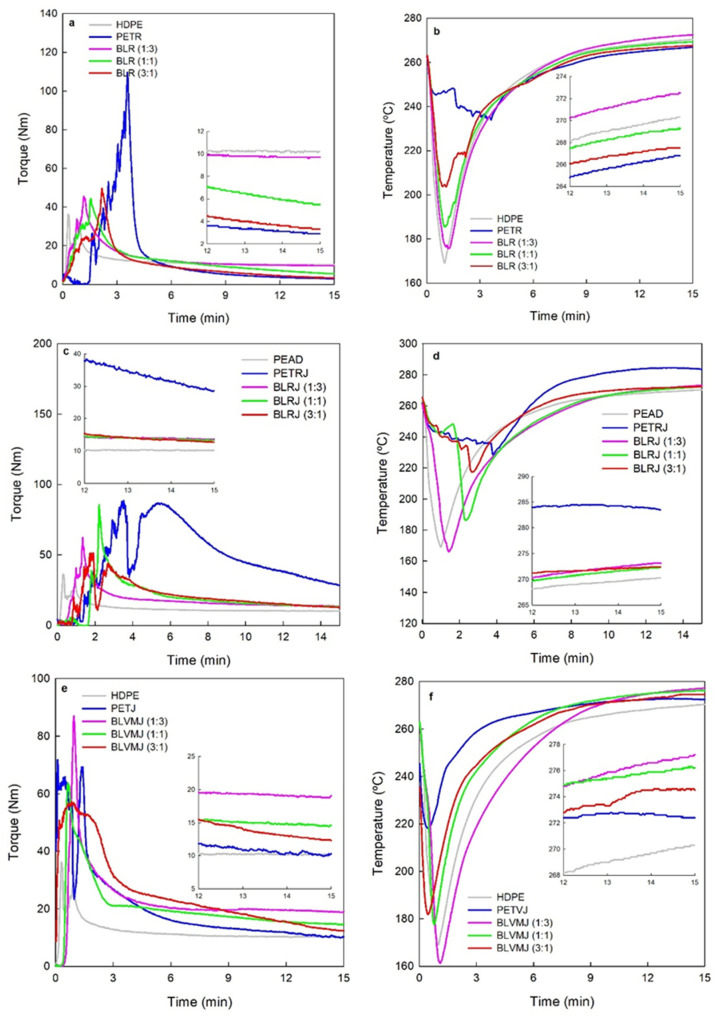
Torque (**a**,**c**,**e**) and temperature (**b**,**d**,**f**) as functions of time for the neat polymers and their blends containing neat (**a**,**b**), additivated (**c**,**d**) and additivated and compatibilized (**e**,**f**) PETR processed at 265 °C, 60 rpm for 15 min.

Figure 1 shows torque and temperature versus time for neat HDPE and PETR as well as for the additivated and the additivated and compatibilized blends. The pure and additivated PETV and PETV/HDPE systems showed a similar behavior.

Melting of PETV, HDPE and the blends with PETV occurred in approximately 3 min of processing causing a decrease in torque. The melting time for PETR and its blends was 6 min, probably due to the larger volume (lower bulk density) of the PETR flakes, which made it difficult to fill the processing chamber.

In the last 3 min of processing (12–15 min), the temperature—around 268 to 270 °C—is nearly independent of composition. Torque, which is proportional to melt viscosity, varies significantly with composition, being almost triple for HDPE (10 Nm) than for PET (3.5 Nm). Torque for the PET/HDPE blend with 75% HDPE is equivalent to that of neat HDPE, whereas the torque for the blend with 75% PET is similar to that of neat PET, reaching an intermediate value (5.5 Nm) in the blend with 50% of each component. No significant differences were observed between the blends with PETV and PETR.

Reproducibility results on the final processing stage on torque and temperature (12 to 15 min) of PETV/HDPE and PETR/HDPE blends showed that the temperature is reproducible in the range ±1 °C to ±2 °C and the torque up to 5 to 15% of the measured value. These are the limits of the accuracy of the results obtained in the present work. In general, there was good reproducibility of the data, and the differences between the torque curves were very small.

**Figure 2:**
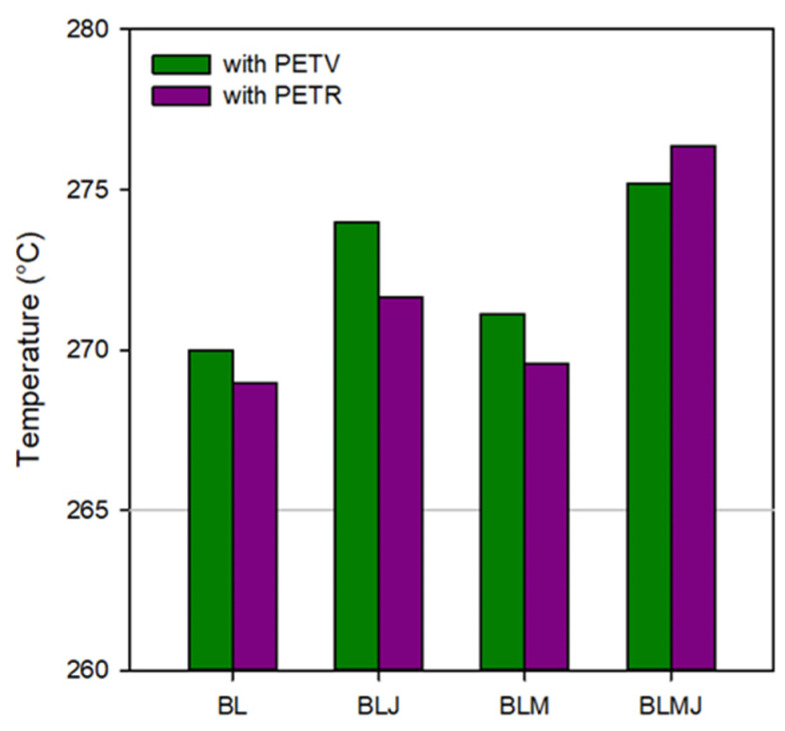
Average temperature in PETV/PEAD (1:1) and PETR/PEAD (1:1) blends at 12 to 15 min interval (BL = pure; BLJ = additivated; BLM = compatibilized; BLMJ = additivated and compatibilized).

Figure 2 shows that the incorporation of both 1 phr Joncryl (chain extender) and 10% PE-g-MA (compatibilizer) resulted in an increase in melt temperature (5–7 °C). The effect of adding solely the compatibilizer is minimal (approximately 1 °C average temperature increase in the last 3 min of processing). However, the addition of the chain extender moderately affects the temperature (3 to 4 °C) in the final stage of processing. This behavior was attributed to the recovery of the system’s molar mass, as the melting temperature in polymers represents the breaking of secondary bonds between the chains and, the longer the chains, the more thermal energy needs to be supplied [20].

**Figure 3:**
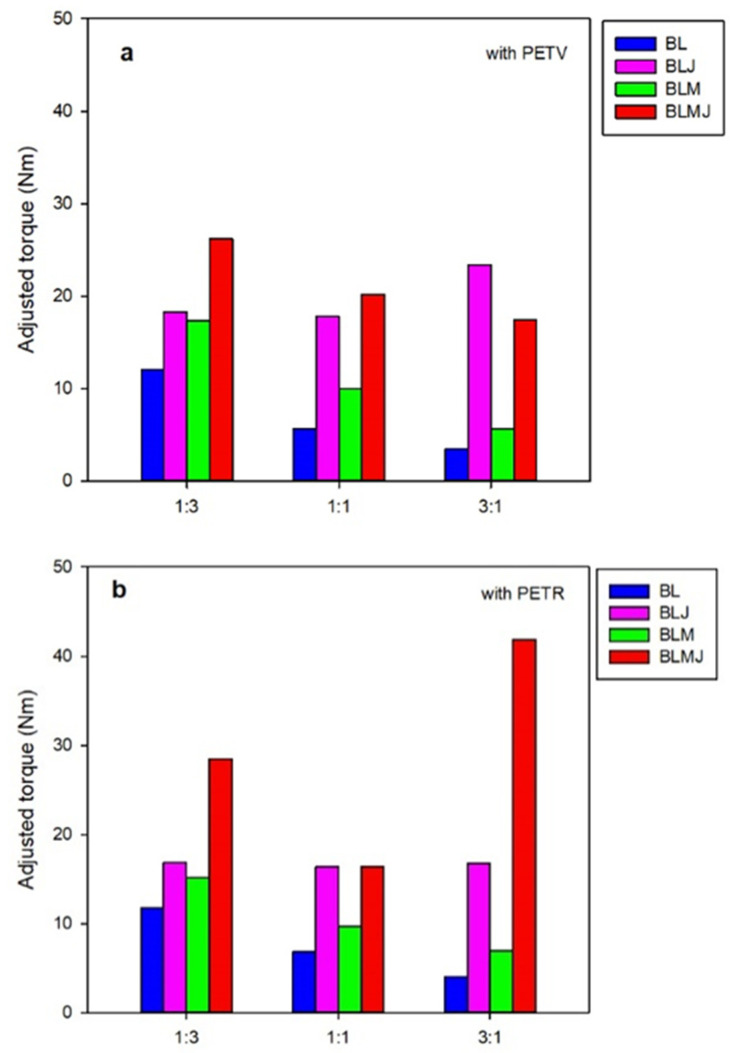
Average adjusted torque in the 12–15 min interval for PETV/HDPE (**a**) and PETR/HDPE (**b**) blends.

Viscosity is a material parameter that is very sensitive to both temperature and weight average molar mass. As mentioned previously, in order to eliminate the dependence of viscosity, and hence torque, on temperature, the adjusted torque $Z^{*}$ is defined (Equation (5)). Figure 3 shows the effect of additivation and compatibilization on the average adjusted torque $Z^{*}$ in the last 3 min of processing (12 to 15 min) evaluated at the reference temperature $T^{*}$ = 265° and a temperature coefficient β = 0.030 °C^−1^ obtained through Equation (2), as well as its difference with the average adjusted torque of the pristine blends $\Delta Z^{*}$.

The results obtained show the strong effect of both additivation and/or compatibilization on the adjusted torque of the blends.

The addition of the compatibilizing agent (10% PE-g-MA) (BLM blends) was found to moderately increase the adjusted torque when compared to additivated and additivated and compatibilized blends. Increases in the range of 30% to 75% relative to the non-compatibilized blends are observed. These increases are practically independent of the blend’s composition (the PET/HDPE ratio and the kind of PET (virgin or recycled)) used.

The incorporation of a small quantity of chain extender (1 phr Joncryl) (BLJ blends) led to torque increases between 50% and 500% compared to the pristine blend. The increases depended on the composition of the blend and increased with PET content, indicating that the additive (chain extender) acts on the polyester and not on the polyolefin, as expected [39]. The effect of the additive was significantly greater in compositions prepared with PETV than in those containing PETR. We believe this difference in behavior to be associated with the lower molecular weight of PETR, as the presence of contaminants in the recycled product should enhance its degradation. Besides, moisture in PETR flakes is higher than in PETV pellets and this should also enhance degradation during processing.

The joint incorporation of compatibilizer and chain extender (BLMJ blends) further increased the viscosity of the system: 125% to 950% compared to the pristine blends. This increase was greater than the sum of the independent effects of the compatibilizer and additive, revealing the synergistic effect between the two. However, the joint effect was greater in the blends with PETR than in those containing PETV. These results clearly show the efficiency of the compatibilizer and the additive in PET/HDPE blends and fully justify the present work.

**Figure 4:**
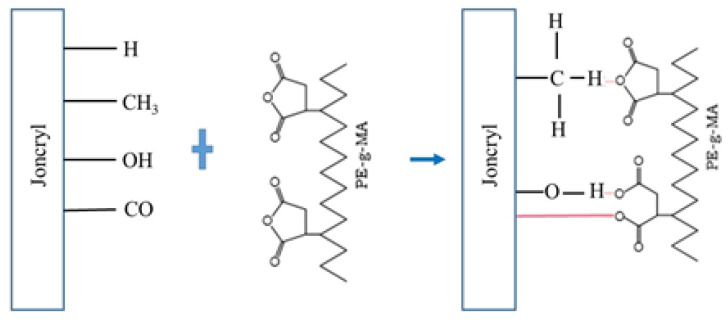
Schematic representation of a possible reaction between Joncryl and PE-g-MA.

Figure 4 shows a hypothetical model of the interaction between the surface of the chain extender (Joncryl) and the compatibilizer (PE-g-MA), revealing that there can be a chemical reaction between the two components and proving their synergism.

#### Degradation and Recuperation during Processing

In addition to the average temperature inside the mixing chamber (°C) and the average set torque (Nm), the rate of change of the set torque (Nm/min) was evaluated by linear regression of $Z^{*}$(t) versus t in the last 3 min of processing. The relative rate of change of the adjusted torque $R_{Z}$ (%/min) and the relative rate of change of the weight average molar mass $R_{M}$ (%/min) were evaluated using the pseudoplasticity index n = 0.8, calculated using Equation (5).

It is worth noting that negative rates correspond to the decrease in viscosity ($R_{z}$) and molar mass ($R_{M}$) with time, i.e., they are measures of the incipient degradation rate of the polymers during the last processing stage. On the other hand, positive rates correspond to the increase in viscosity and molar mass with time and reveal the recovery of physical properties of the melt possibly due to chain extension, branching and/or cross-linking of the polymer chains. Experience indicates that only values of |$R_{M}$| > 1 %/min can be considered positive experimental evidence of degradation or recovery, and smaller values can be included in the uncertainty [36].

**Figure 5:**
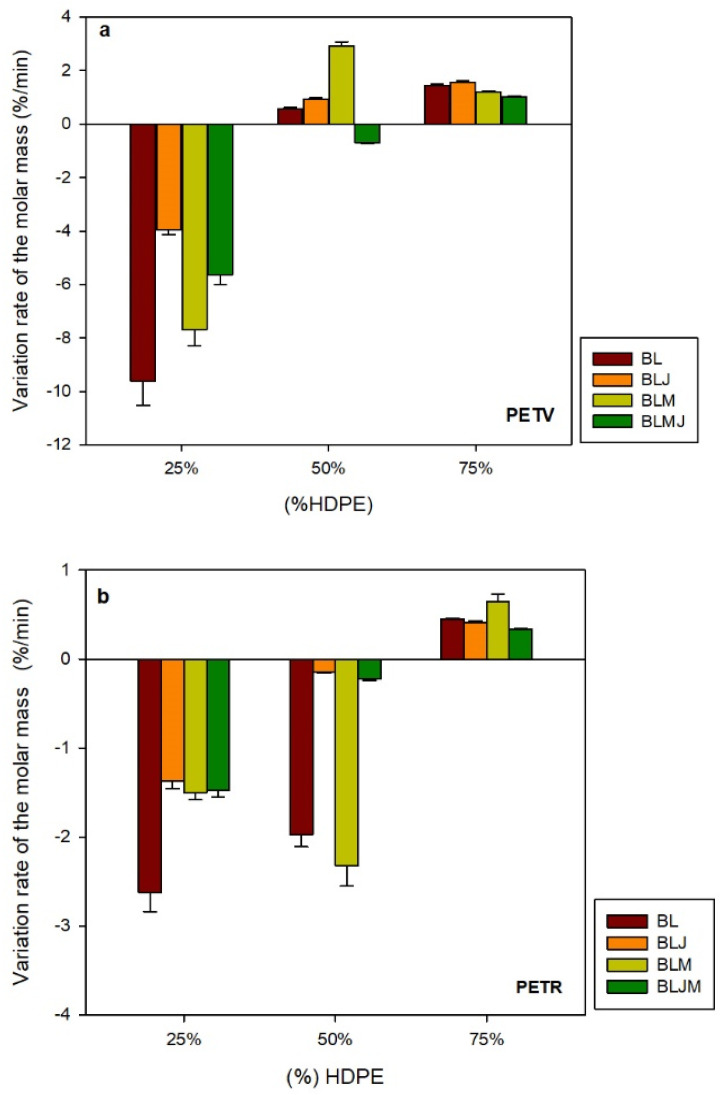
Relative rate of change of the weight average molar mass of different components as a function of HDPE content, over the same time interval. (**a**) PETV and (**b**) PETR.

Figure 5 shows the relative rate of change of the weight average molar mass in the last minutes of processing in the internal mixer, for the pure components and for the pristine (BL), additivated (BLJ), compatibilized (BLM) and for the additivated and compatibilized (BLMJ) blends.

Figure 5 shows a moderate degradation of the neat blends (BL) with higher PET content and the absence of degradation in the blends with higher HDPE content. Regarding the type of PET (virgin versus recycled), no major differences were observed, except for the blend with 50% of each component, which presents an exceptional character: higher viscosity in the blend with PETV and degradation during processing in the blend with PETR. The reason for this behavior is not clear at the moment and might be associated with differences in the moisture content of these blends or to differences in morphology. Further in-depth studies need to be performed in order to address this issue and to explain this phenomenon very clearly.

A comparison of the blends containing Joncryl (Figure 3), indicates a significant increase in viscosity—almost double—and a slight decrease in the degradation rate during processing (Figure 5) in PET and blends containing 75% PET, thus proving the efficiency of this additive towards PET. Guclu [40] investigated the effect of using a multifunctional epoxide chain extender (Joncryl^®^ ADR 4468) on the thermal stabilization and rheological properties of recycled polyethylene terephthalate and its blends with polybutylene terephthalate, and the reactivity of Joncryl was more noticeable in blends with higher recycled polyethylene terephthalate contents due to the higher available internal reactive sites of much shorter recycled polyethylene terephthalate molecules.

Comparison of the compatibilized blends with pristine blends yields a significant increase in viscosity (30% to 100%) with compatibilization and no significant variation in degradation during processing. A similar result was obtained by Jarukumjorn et al. [41] when they studied the rheological behavior of recycled HDPE blends with virgin PET using a capillary rheometer. The authors observed that the viscosities of the blends decreased as the HDPE component in the blends decreased. The viscosities of the PE-g-MA compatibilized blends (2, 5, 7, 10 phr) were higher than the non-compatibilized blends and increased with the amount of compatibilizer. The increase in viscosities is attributed to greater interaction between the components of the blends.

Both additivation and compatibilization result in quite significant increases in the viscosity of the blends, as already indicated (Figure 3). The simultaneous incorporation of Joncryl and PE-g-MA appears to have a synergistic effect on viscosity, leading to increases greater than those expected from an additive behavior. No significant variations in degradation were observed during the processing of the blends that can be attributed to the joint effect of the additive and the compatibilizer.

Comparison of all available results reveals that, with regard to incipient degradation during processing, only PET and PET-rich blends show significant values, -RM > 1 %/min, as shown in Table 2. Degradation during processing is rather moderate in all cases. Both additive and compatibilizer incorporation decrease degradation discretely and recycled PET is more sensitive to degradation than virgin PET.

Some authors [20,26] have suggested two explanations for the observed behavior: (a) that the chain extender (Joncryl) added to the blends acted as a lubricant and that more severe processing conditions would be needed to fully disperse this additive in the blend and (b) that PETR degrades more during processing than PETV.

### 3.2. DSC Thermal Analysis: Melt Crystallization

**Figure 6:**
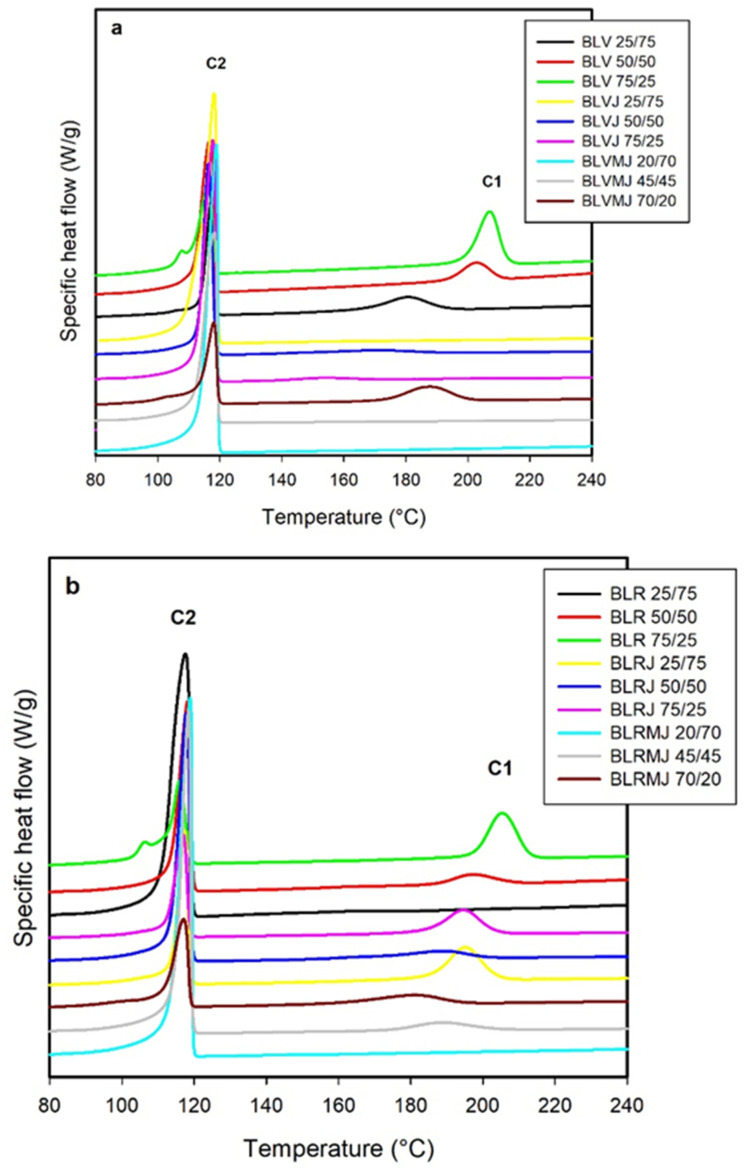
Heat flow as a function of temperature for PETV/HDPE (BLV) (**a**) and PETR/HDPE (BLR) (**b**) blends with 1:3, 3:3, 3:1 ratios, additivated (J) and compatibilized and additivated (MJ), crystallized from the melt with crystallization peaks (C), identified for PET (1) and HDPE (2).

Crystallization from the melt during cooling are reported as heat flux (J) versus time (t). Separate peaks for the crystallization of each component were observed in the blends, as illustrated in Figure 6, for PET/HDPE blends with virgin or recycled PET at various concentrations and additivated with Joncryl or with a combination of Joncryl and compatibilizer.

In Figure 6, the phase change events are coded as shown below: C1: PET crystallization during cooling; C2: HDPE crystallization during cooling.

As with torque rheometry, the influence of introducing the recycled material into the mixtures, virgin PET samples were compared with the corresponding recycled materials. No cold crystallization events were observed during heating, indicating that the PET completely crystallized in the processed samples.

On the other hand, it was observed that HDPE crystallized rapidly at around 115 °C exhibiting a crystallinity of 60% and that PET crystallized more slowly at around 205 °C exhibiting a crystallinity of 25 to 30%. No significant differences in crystallization were observed between virgin and recycled PET. The incorporation of 1 phr Joncryl hindered PET crystallization, which crystallized at 22 °C (PETV) and 14 °C (PETR) lower temperatures than the pure polymer and also exhibited a 4 to 5% lower crystallinity. This may have occurred due to the interaction of the additive with PET and to a lower activation energy of the system.

The crystallization parameters from the melt presented in Table 3 for the neat, additivated and additivated and compatibilized blends suggest that the differences in morphology did not affect HDPE crystallization but rather PET crystallization—particularly in the case where the PET content (virgin or recycled) was the smallest (25%).

The HDPE in the blends crystallizes as a sharp and moderately asymmetric peak (event C2). The type of PET (V or R) or the incorporation of the additive (Joncryl) or of the additive and compatibilizer (Joncryl + PE-g-MA) did not significantly affect HDPE crystallization. HDPE crystallization temperature decreases from 118 °C (neat HDPE) to 108 °C (blend with 75% PETV) or 106 °C (blend with 75% PETR): a significant drop of 10 to 12 °C. The degree of HDPE crystallinity in the blend was not significantly affected by the presence of PET or chain-extending additive (Joncryl). However, the incorporation of compatibilizer (PE-g-Ma) clearly increased the crystallinity and this effect was more significant on the 1:3 blends, i.e., those with 75% HDPE.

PET in the blends crystallizes in a wider and more symmetrical peak than HDPE (event C2) at temperatures around 200 °C when the HDPE content is 0 to 50%, dropping to 150 °C in the blends with 25% PETV and 165 °C in the blends with 25% PETR: a very significant 35 to 50 °C drop. The crystallinity remains around 30% in the blends with PETV but suffers a significant and progressive decrease with increasing HDPE content in the blends with PETR, reaching 2% crystallinity in the blends with 75% HDPE. These discrepancies in crystallization behavior between PET and PETR are well-known in the literature, being attributed to the different origins of these materials; in the case of PETR there is the presence of different crystalline phases resulting from the contamination of this waste [7,42,43,44].

The additivation (1 phr Joncryl) greatly affects the crystallization of PET (event C1). In samples prepared with virgin PET, decreases in peak crystallization temperature of between 20 and 50 °C for the different blends were observed while the degree of crystallinity decreased, reaching values of 15 to 35%, which can be attributed to a significant chain extension reaction with the products hindering PET crystallization. In the samples prepared with recycled PET, a similar effect is observed but of less intensity: the peak temperature decreases by 10 °C and crystallinity by 5%, approximately.

The incorporation of Joncryl into the HDPE/PET blend promotes an increase in molecular weight and the formation of cross-linking points increases the difficulty of organization and aggregation of the PET chains. Thus, a higher activation free energy is required for the formation of the crystalline phase as well as the diffusion of PET chains toward the crystal surface during cooling, and this will actually decrease the nucleation rate of PET. As a result, the crystallization peak corresponding to PET shifts to a lower temperature, and the crystallization of PET decreases in the presence of the chain extender additive. A similar result was observed by Zhang et al. when a polymeric methylene diphenyl diisocyanate PMDI was added to R-PET/LLDPE/SEBS-g-MA blends [45].

PE-g-MA compatibilization resulted in the absence of PET crystallization (C1) in the blends (1:3) and (1:1) PETV/HDPE and in PETR/HDPE (1:3) blends. The simultaneous effect of additivation and compatibilization in the blends resulted in a crystallization behavior similar to that observed for the compatibilized blends. Bae et al. [46] reported that the disappearance of the PET peak in the crystallization during cooling suggests that the temperature of PET crystallization coincided with the usual TC of PP, due to the chemical bonding product between the dispersed PET phase and the functionalized polyolefin. In fact, it is possible that the grafted copolymer located at the interface may act as a polymeric diluent to retard PET crystallization. Matias et al. [47] obtained similar results for the mixture of recycled PET and PP and reported that the presence of compatibilizer affects PET crystallization.

Tariq et al. [48] found similar results when studying the properties of blends of polyethylene terephthalate (PET) and polypropylene (PP) compatibilized with PP functionalized with maleic anhydride (PP-g-MAH). The authors reported that PET crystallinity was low due to the impediment created to the formation of crystals by the interactions generated between PP and PET after the addition compatibilizer.

### 3.3. TGA Thermal Analysis

**Figure 7:**
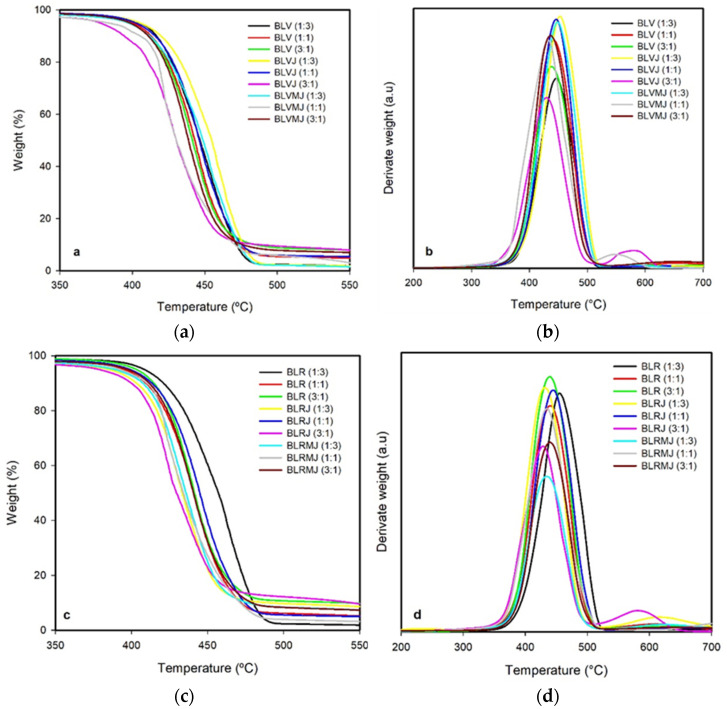
(**a**–**c**) Thermogravimetric (TGA) and (**b**–**d**) TGA derivative (DTG) curves for the neat, additivated and compatibilized and additivated PETV/HDPE and PETR/HDPE blends.

The thermal stability of the neat components, HDPE, PETV and PETR, the additive Joncryl and the compatibilizer PE-g-MA, as well as that of the PETV/HDPE and PETR/HDPE blends, with and without the incorporation of the additive or both the additive and compatibilizer were investigated by TGA. The TGA and DTG curves for the additivated and additivated and compatibilized (MJ) blends are presented in Figure 7.

In all cases, between the temperatures of 100 °C and 200 °C there was a small initial mass loss (1 to 2%) attributable to the moisture present in the samples. PETV and PETR samples showed similar results, with 85% mass loss between 365 and 480 °C and with total (extrapolated) loss between 750 and 850 °C. HDPE and PE-g-MA showed similar results, with 95% mass loss between 404 and 480 °C and total mass loss at 600 °C. Joncryl, the oligomer, lost 90% of mass between 230 °C and 460 °C, with total mass loss observed at 600 °C.

Replacing virgin PET with recycled PET did not cause significant changes in the degradation profile of the PET/HDPE blends. Simultaneous compatibilization and additivation also did not change the stability of the blends.

From the DTG profiles, a broad peak corresponding to a single degradation process is observed for maximum mass loss for all PET/HDPE blends. Chen et al. [49] also observed only one peak in the DTG curve at 446 °C for the recycled HDPE/PET blend. The authors suggested that the blend is composed of a series of interconnected monomers, thus allowing a temperature increase to promote random chain scission by thermal degradation and depolymerization occurring at weak sites in the polymer chains.

The higher amount of residual mass found in samples containing PETR is consistent with the presence of inorganic filler, an additive currently used in the processing of commercial-grade polymers [50]. This is a strong indication that the replacement of virgin material with residual polymeric residue should not impair the thermal degradation of HDPE/ PET blends.

## 4. Conclusions

The present work follows the current trend on the study and analysis of post-consumer plastic waste, aiming to improve its properties from a technological point of view and favoring its wider use. The data obtained indicates that the incorporation of 1% of a chain extender additive (Joncryl) and 10% of a compatibilizer (PE-g-MA) promoted a considerable increase in the melt viscosity (and hence torque) of PETV, and this increase was even higher for PETR, especially in the presence of the chain extender. This increase occurred for both blends, regardless of the composition and kind of PET used. PE-g-MA compatibilized blends also showed a considerable elevation in torque which increased with the amount of HDPE in their composition, regardless of the kind of PET (virgin or recycled) used. Torque was considerably higher for the blends additivated and compatibilized when compared with those that were only compatibilized or only additivated, proving that there is an interaction between the additive and the compatibilizer. Although blend degradation rate during processing increases with PET content, it is smaller in PETR/HDPE blends compared to PETV/HDPE blends. Joncryl incorporation hinders the crystallization of both PET and PET/HDPE blends but does not affect the crystallization of HDPE. The addition of PE-g-MA minimally affects the crystallization of HDPE in the blend; however, it significantly alters the crystallization of PET. The combined incorporation of the additive (Joncryl) and compatibilizer (PE-g-MA) only affects PET crystallization. The thermal stability of the blends is similar to that of neat PET. Incorporation of both additive and compatibilizer does not alter the thermal stability of the blends.

Our data indicates that it is possible to develop polyolefin (HDPE)/ polyester (PET) blends and to promote increases in melt strength and torque recovery with proper additivation. The efficiency of PE-g-MA and Joncryl in the compatibilization of this mixture was proven.

## Figures and Tables

**Table 1:** Samples’ compositions and codes.

Code	Mass (g)					Total
	PETV	PETR	HDPE	Joncryl	PE-*g*-MA	
PETV	301	-	-	-	-	301
PETR	-	301	-	-	-	301
PETVJ	301	-	-	3	-	304
PETRJ	-	301	-	3	-	304
HDPE	-	-	207	-	-	207
BLV (1:3)	61	-	181	-	-	242
BLR (1:3)	-	61	181	-	-	242
BLV (1:1)	121	-	121	-	-	242
BLR (1:1)	-	121	121	-	-	242
BLV (3:1)	181	-	61	-	-	242
BLR (3:1)	-	181	61	-	-	242
BLVJ (1:3)	61	-	181	2.45	-	245
BLRJ (1:3)	-	61	181	2.45	-	245
BLVJ (1:1)	121	-	121	2.45	-	245
BLRJ (1:1)	-	121	121	2.45	-	245
BLVJ (3:1)	182	-	61	2.45	-	245
BLRJ (3:1)	-	182	61	2.45	-	245
BLVM (1:3)	53	-	187	-	24	266
BLRM (1:3)	-	53	187	-	24	266
BLVM (1:1)	119	-	119	-	24	264
BLRM (1:1)	-	119	119	-	24	264
BLVM (3:1)	187	-	53	-	24	266
BLRM (3:1)	-	187	53	-	24	266
BLVMJ (1:3)	53	-	187	3	24	269
BLRMJ (1:3)	-	53	187	3	24	269
BLVMJ (1:1)	119	-	119	3	24	267
BLRMJ (1:1)	-	119	119	3	24	267
BLVMJ (3:1)	185	-	53	3	24	267
BLRMJ (3:1)	-	185	53	3	24	267

**Table 2:** Parameter: R_M_ (%/min).

Composition	PETV	PETR	BLV (3:1)	BLR (3:1)	BLR (1:1)
Pristine	---	1.8	2.9	2.6	2.0
Additivated (Joncryl)	1.6	3.1	1.2	1.4	---
Compatibilized (PE-g-MA)	---	1.8	2.3	1.5	2.3
Additivated + Compatatibilized (Joncryl + PE-g-MA)	1.6	3.1	1.7	1.5	---

---: did not have significant degradation (R_M_ < 1%).

**Table 3:** Crystallization parameters of the neat, additivated, compatibilized and additivated and compatibilized blends.

	Composition (HDPE/PET)	Blends with PETV						Blends with PETR					
		T_c1_ (°C)	T_c2_ (°C)	ΔH_c1_ (J/g)	ΔH_c2_ (J/g)	ΔX_c1_ (%)	ΔX_c2_ (%)	T_c1_ (°C)	T_c2_ (°C)	ΔH_c1_ (J/g)	ΔH_c2_ (J/g)	ΔX_c1_ (%)	ΔX_c2_ (%)
Pure	1:3	207.1	107.8	41.6	159.1	29.7	54.3	205.4	106.4	41.8	164.7	29.9	56.2
	1:1	202.6	116.8	39.2	164.6	28.0	56.2	197.2	118.2	26.3	166.6	18.8	56.9
	3:1	150.7	117.6	24.9	172.6	36.1	58.9	165.0	117.5	2.9	168.5	2.0	59.0
1% Joncryl	1:3	153.5	107.8	7.1	165.7	5.1	56.6	194.6	117.0	37.6	168.4	26.8	57.5
	1:1	170.6	117.2	17.2	163.6	12.2	55.8	188.6	117.8	31.5	168.9	22.5	57.6
	3:1	151.1	118.1	1.8	164.6	1.3	56.2	195.1	117.3	112.4	52.9	80.1	18.7
10% Pe-g- MA	1:3	198.7	106.0	43.9	244.3	31.4	83.4	198.9	105.6	42.2	224.7	30.2	76.7
	1:1	---	117.7	---	180.4	---	61.6	188.4	118.0	21.8	188.5	15.6	64.3
	3:1	---	117.8	---	188.2	---	64.2	---	118.4	---	174.1	---	59.4
Joncryl+ Pe-g- MA	1:3	187.6	118.0	34.6	229.0	24.7	78.2	181.0	117.1	26.6	228.8	19.0	78.1
	1:1	---	118.2	---	199.7	---	68.2	188.7	118.4	25.4	205.2	18.2	70.0
	3:1	---	118.9	---	196.5	---	67.1	---	119.1	---	186.4	---	63.6

## Data Availability

The data that support the findings of this study are available upon request from the authors.
